# COVID-19 in 28-Week Triplets Caused by Intrauterine Transmission of SARS-CoV-2—Case Report

**DOI:** 10.3389/fped.2021.812057

**Published:** 2021-12-24

**Authors:** Sigrid C. Disse, Tatiana Manuylova, Klaus Adam, Annette Lechler, Robert Zant, Karin Klingel, Christian Aepinus, Thomas Finkenzeller, Sven Wellmann, Fritz Schneble

**Affiliations:** ^1^Children's Hospital Weiden, Kliniken Nordoberpfalz Aktiengesellschaft (AG), Weiden, Germany; ^2^Institute of Pathology and Neuropathology, University Hospital Tübingen, Tübingen, Germany; ^3^Women's Hospital Weiden, Kliniken Nordoberpfalz Aktiengesellschaft (AG), Weiden, Germany; ^4^Institute of Laboratory Medicine and Microbiology, Synlab Medizinisches Versorgungszentrum (MVZ) Weiden, Weiden, Germany; ^5^Institute of Radiology, Hospital Weiden, Kliniken Nordoberpfalz Aktiengesellschaft (AG), Weiden, Germany; ^6^Department of Neonatology, University Children's Hospital Regensburg (KUNO) at the Hospital St. Hedwig of the Order of St. John, University of Regensburg, Regensburg, Germany

**Keywords:** vertical transmission, case report, triplets, neonate, SARS-CoV-2, preterm, COVID-19

## Abstract

Since the beginning of the COVID-19 pandemic, in-utero transmission of SARS-CoV-2 remains a rarity and only very few cases have been proven across the world. Here we depict the clinical, laboratory and radiologic findings of preterm triplets born at 28 6/7 weeks to a mother who contracted COVID-19 just 1 week before delivery. The triplets showed SARS-CoV-2 positivity right after birth, developed significant leukopenia and early-onset pulmonary interstitial emphysema. The most severely affected triplet I required 10 days of high-frequency oscillatory ventilation due to failure of conventional invasive ventilation, and circulatory support for 4 days. Despite a severe clinical course in two triplets (triplet I and II), clinical management without experimental, targeted antiviral drugs was successful. At discharge home, the triplets showed no signs of neurologic or pulmonary sequelae. Placental immunohistology with SARS-CoV-2 N-protein localized strongly to syncytiotrophoblast cells and, to a lesser extent, to fetal Hofbauer cells, proving intrauterine virus transmission. We discuss the role of maternal viremia as a potential risk factor for vertical transmission. To the best of our knowledge, our report presents the earliest unequivocally confirmed prenatal virus transmission in long-term surviving children, i.e., at the beginning of the third trimester.

## Introduction

Whether vertical transmission (VT) is a possible route of SARS-CoV-2-infection in neonates has intrigued and worried medical specialists and families worldwide. After 2 years of global COVID-19-pandemic with >247 million *cumulative cases* of infected people worldwide (1), reports on SARS-CoV-2 infections in preterm neonates are still scarce and the few published data suggest a mild to moderate course in neonates (2–5). Thus, we want to share the unique course, laboratory and radiological findings in a set of triplets with the scientific community. We report (1) a severe course of COVID-19 in two triplets, and (2) histopathologic analysis of the triplets' placenta, showing in utero-transmission of SARS-CoV-2.

## Case Description

**Figure 1:**
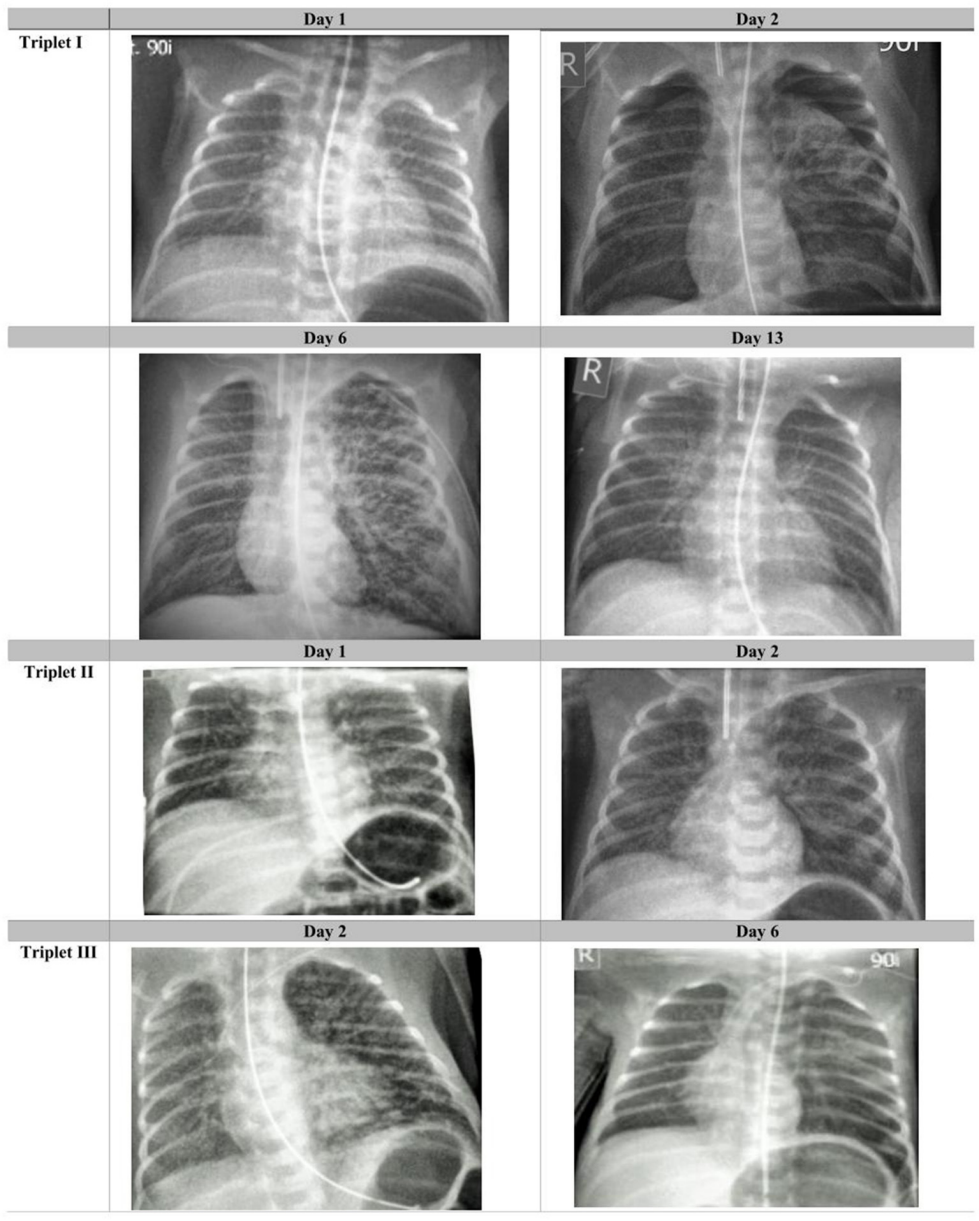
Radiological course in the triplets: Chest radiographies taken on days 1, 2, 6, and 13 after birth in triplet I; on days 1 and 2 in triplet II and on days 2 and 6 in triplet III (selected radiographs shown). Images are windowed in order to optimize visualization of the relevant findings.

A previously healthy Caucasian 36-year-old gravida 2 para 1 at 28 3/7 gestational weeks, had noted moderate respiratory symptoms for a few days and first tested positive for SARS-CoV-2 at a public test center (PCR test, EcoCare test center, Weiden). She presented to our Women's Hospital due to fever few days later, where the infection was confirmed (PCR test from nasopharyngeal swab; Allplex 2019-nCov, Seegene, Seoul, Korea*)*. The pregnancy was intact with trichorionic triplets, conceived after intrauterine insemination. Maternal observation was uneventful, and she was administered two doses of steroids for fetal pulmonary maturation. She was discharged home > 24 h after administration of the second dose of steroids in a stable state. Few hours later, premature rupture of membranes occurred, and urgent C-section was performed at 28 6/7 weeks- 8 days after the mother first tested positive for SARS-CoV-2. The triplets were delivered without complications and adaptation was unproblematic in all three (triplet I: male–birthweight 1,150 g, APGAR scores 8/9 and 9 at 1, 5, and 10 min; triplet II: female−990 g, APGAR scores 8/9 and 9; triplet III: female- 930 g, APGAR scores 7/9 and 9). Physical examination showed eutrophic preterm newborns without any externally visible anomalies. They were referred to our neonatal intensive care unit (NICU) on non-invasive intermittent positive pressure ventilation (NIPPV) in closed incubators, requiring none or low concentrations of supplementary oxygen, and received standard neonatal care. Following strict hygiene precautions, both parents were only allowed to have any contact to the children after they tested negative twice, and until symptoms of COVID-19 had resolved completely. Calculated antibiotic therapy was initiated directly after delivery; the blood cultures remained sterile. The children showed progressive leukopenia but low CrP and IL-6 values (Table 1). RT-PCR assays for SARS-CoV-2 were performed from nasopharyngeal swabs immediately after birth, showing positive results in triplet I and III, and repeatedly thereafter (Table 1). In triplet II, PCR from tracheal aspirate on day of life (DOL) 2 also confirmed an infection with SARS-CoV-2. The triplets developed signs of progressive respiratory distress within few postnatal hours. Approx. 30 h after birth, triplets I and II were intubated and placed on mechanical ventilation. Intratracheal administration of surfactant (100 mg/kg poractant alfa, Curosurf^®^) resulted in only moderate improvement. Few minutes after intratracheal instillation of surfactant in triplet I, chest radiography was available, showing bilateral pneumothorax which had presumably been present shortly prior to surfactant administration. A 0.8 Fr. chest tube was inserted into the left anterior pleural space for drainage. In all triplets, chest radiographies showed bilateral, diffuse, multifocal cystic and linear radiolucencies and signs of lung hyperinflation, clearly distinct of prematurity-related acute respiratory distress syndrome (pmARDS) but resembling pulmonary interstitial emphysema (Figure 1). Triplet I was switched to high-frequency oscillation ventilation (HFOV) shortly after intubation due to failure of conventional invasive ventilation and required high concentrations of supplementary oxygen (Table 1). For circulatory support, he received dopamine intravenously for DOL 2-6. Due to stable conditions in all three, we decided against experimental administration of targeted antiviral drugs. Triplet I was extubated successfully on DOL 15 following administration of a single dose of dexamethasone (0.1 mg/kg). Triplet II required continuous positive airway pressure (CPAP) support via high-flow nasal cannulas with ambient air until a corrected age of 36 4/7 weeks. Triplet I and II required intravenous ibuprofen for closure of a hemodynamically relevant patent ductus arteriosus. Serial cranial ultrasounds were normal in all children. Enteral feeding was initiated with formula for 4 days, then pasteurized maternal breast milk was available. A sample of breast milk tested negative for SARS-CoV-2, using RT-PCR. The triplets showed no signs of feeding intolerance. We discharged them at a corrected gestational age of 38 weeks, breathing spontaneously and without neurological symptoms.

**Table 1:** Overview of the results from PCR assays on SARS CoV-2 WBC count, CrP and need for respiratory support on selected days.

	**Postpartum**	**DOL 2**	**DOL 3**	**DOL 5**	**DOL 7**	**DOL 10**	**DOL 14**	**Week 3**	**Week 4**
								**(any day)**	**(any day)**
**Laboratory characteristics: PCR-based assays on SARS-CoV-2. Specimen: pharyngeal swab (ps) or tracheal aspirate (ta)**.						
Triplet 1 (male; 1,150 g)	pos. (ps)	n.inv.	2 x pos.: 1 (ps), 1 (ta)	pos. (ps)	n.inv.	pos. (ta)	pos. (ta)	pos. (ps)	neg. (ps)
Triplet 2 (female, 990 g)	neg. (ps)	1 neg (ps), 1 pos. (ta)	n.inv.	pos. (ps)	n.inv.	pos. (ps)	pos. (ps)	neg. (ps)	pos. (ps)
Triplet 3 (female, 930 g)	pos. (ps)	pos. (ps)	n.inv.	neg. (ps)	n.inv.	neg. (ps)	neg. (ps)	neg. (ps)	neg. (ps)
**Laboratory characteristics: WBC count (G/l), C-reactive Protein (mg/l)**						
Triplet 1: WBC	6.6	2.6	2.1	1.9	n.inv.	8.7^*^	13.1	15.0	15.5
CrP	<0.3	1.5	0.7	0.6		3.3	0.6	0.4	<0.3
Triplet 2: WBC	7.4	QNS	n.inv.	3.7	5.9	n.inv.	n.inv.	24	23.2^**^
CrP	<0.3	0.8		<0.3	0.5		0.7	<0.3	0.6
Triplet 3: WBC	5.6	4.4	4.0	4.8	7.3	n.inv.	12.2	15.9	6.9
CrP	0.3	QNS	1.3	0.9	2.1		2.9	<0.3.	55^***^
**Mechanical ventilation/respiratory support**						
Triplet 1: type	NIPPV	SIMV → HFOV	HFOV	HFOV	HFOV	HFOV	SIMV	CPAP → NIPPV	CPAP
FiO_2_	0.21 → 0.38	0.6	0.5	0.35	0.35	0.30	0.23	0.21 → 0.5^****^	0.21
Triplet 2: type	NIPPV	SIMV	SIMV	SIMV → NIPPV	NIPPV	CPAP	CPAP	CPAP	CPAP
FiO_2_	0.25 → 0.5	0.6 → 0.25	0.25	0.25 → 0.3	0.25	0.21	0.21	0.21	0.21
Triplet 3: type	NIPPV	NIPPV	NIPPV	NIPPV	NIPPV → CPAP	CPAP	CPAP	CPAP	CPAP
FiO_2_	0.21 → 0.3	0.3	0.38	0.21	0.21	0.21	0.21	0.21	0.21

*For weeks 3 and 4, the most pathologic finding is displayed, e.g., “CPAP,” if needed on any day during the respective week*.

*Abbreviations: CPAP, continuous positive airway pressure; HFOV, high-frequency oscillatory ventilation; NIPPV, non-invasive positive pressure ventilation; ps, pharyngeal swab; QNS, quantity not sufficient; SIMV, synchronized intermittent mandatory ventilation; ta, tracheal aspirate; WBC count, white blood cell count; DOL, day of life. Annotations*:

* *After blood transfusion*.

** *DOL 25-clinical signs of bacterial infection with bradycardia and desaturations, interleukin-6 178 pg/ml, blood cultures showed Acinetobacter pittii, the patient received antibiotic treatment with piperacillin/tazobactam and teicoplanin for 7 days. The blood culture taken postnatally remained sterile*.

*** *DOL 26-clinical signs of bacterial infection with bradycardia, the patient received treatment with Meropenem and Teicoplanin, blood cultures taken before treatment remained sterile; throat swabs identified Enterobacter faecium*.

**** *DOL 17-following a phase of stability requiring only CPAP (FiO_2_ 0.21–0.23), secondary respiratory deterioration due to increase in masses of solid mucus, but no hint toward bacterial infection*.

## Diagnostic Assessment

### Laboratory Workup

Analysis of maternal blood taken 5 days after delivery showed a substantial viral load (Ct-value 34.5, estimated ~100–1,000 GE/ml, Ridagene SARS-CoV-2, R-biopharm, Darmstadt, Germany), despite a strong Immunoglobulin A immune response (IgA-Ratio >10; positive >1.1) and positive IgG immune response (IgG-ratio 1.9; positive > 1.1, Euroimmun, Lübeck, Germany). RT-PCR from the serum of the triplets (100 ul serum each) could not detect SARS-CoV-2 specific RNA in the blood, as a possible sign of systemic infection. Blood samples taken after birth in the triplets showed no hint toward an already detectable IgG response transmitted via the placenta (triplets I-III, IgG, all index ratios <0.1 respectively). Ten weeks after birth, triplet I and III showed medium to high SARS-CoV-2 specific IgG but only low to no IgA response (Triplet I IgG 3.9, IgA 1.11, triplet III IgG 6.1, IgA 0.4). Triplet II, with severe clinical course, had no SARS-CoV-2 specific immune response at this time point (IgG 0.7; IgA 0.4). Surprisingly, viral load and the duration of virus persistence showed a strong association with disease severity: In triplet II, five nasopharyngeal swabs or tracheal aspirate specimens tested positive during the hospital stay, for the last time on DOL 25; among the five positive tests, two showed Ct-values <30 indicating a high viral load. Triplet I had seven positive tests, among which six showed Ct values <30, while triplet III had only two positive tests on DOL 1 and 2, both with low viral load.

### Pathology Workup

**Figure 2:**
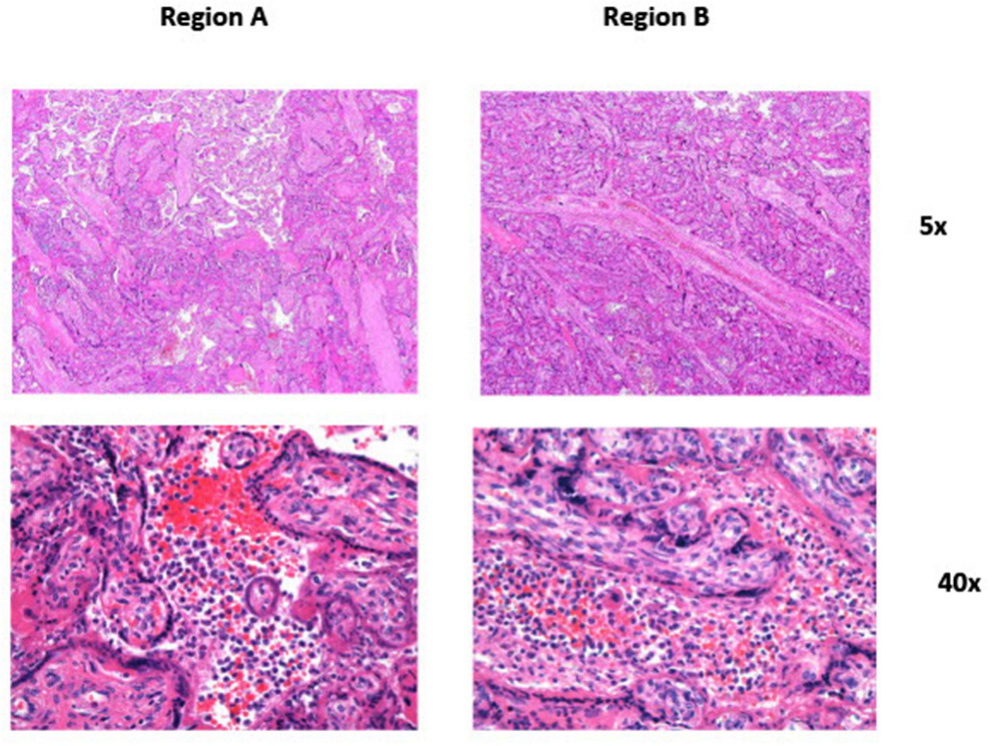
Panel 1 (Region A_5x): Focal chronic histiocytic intervillositis is seen in the center of the picture. Panel 2 (Region A_40x): Higher magnification of the center of Panel 1 showing intervillous histiocytic aggregates. Panel 3 (Region B_5x): Focal chronic histiocytic intervillositis is seen in the center of the picture. Compared to Panel 1 the changes seem a bit more pronounced. Panel 4 (Region B_40x): Higher magnification of the center of Panel 3 showing intervillous histiocytic aggregates.

Macroscopic examination showed trichorionic triamniotic fused placentas, weighing 770 g and measuring 26 × 28 × 3 cm. The cut surface of the placenta as well as the amnion were unremarkable. Histologically, placental villi revealed different maturation status, discrete intervillous fibrin deposition (Figure 2), and focal degeneration of the villi. No massive intervillous fibrin depositions were noted. In some intermediate and stem villi, occlusive changes in the vessels were obvious. Additionally, focal intervillous histiocytic aggregates were seen, compatible with chronic histiocytic intervillositis (Figure 2). These aggregates showed minimal regional differences, in line with the different viral loads of the fetuses.

**Figure 3:**
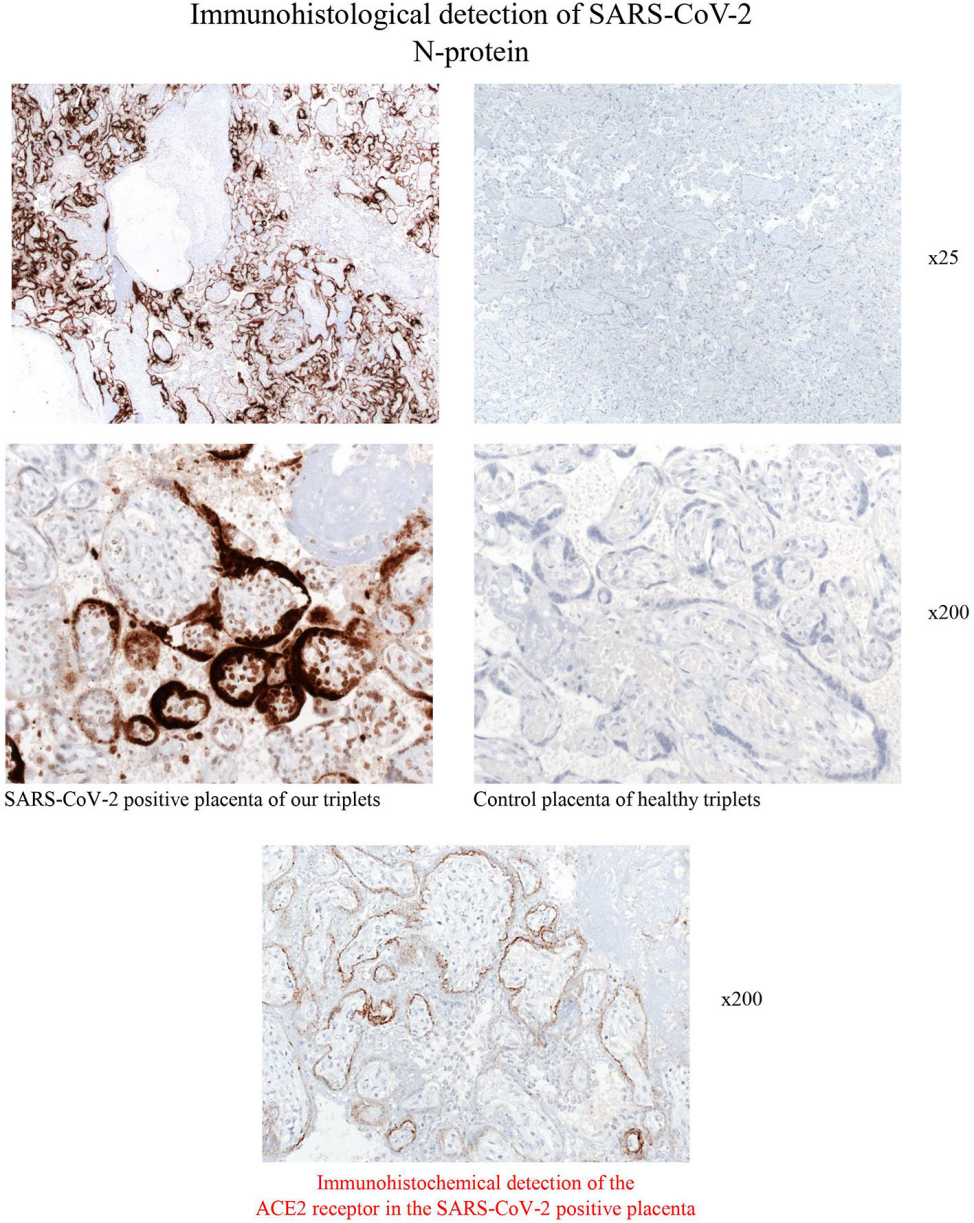
Immunohistological stainings for the detection of SARS-CoV-2- N-protein of the placenta of the COVID-19 positive triplets. Syncytiotrophoblast cells reveals strong membrane positivity for the viral N-protein. Scattered positive macrophages, Hofbauer cells and stroma cells are also virus positive. Control placenta of healthy triplets was consistently negative in immunohistochemistry (magnification x25 and x200).

Immunohistology analysis of the triplets' placenta after discharge was performed as follows: For the detection of SARS-CoV-2 N-protein, 4 um thick tissue sections of paraffin embedded placentas (including normal placentas) we used the monoclonal mouse anti-SARS-CoV-2 N-protein antibody (clone N-IH-H5, 1:200, Mediagnost, Reutlingen, Germany). Immunohistochemical analysis was performed on an automated immunostainer following the manufacturer's protocol (Benchmark; Ventana Medical Systems, Tucson, AZ) and using the ultraView detection system (Ventana) and diaminobenzidine as substrate. Tissue sections were counterstained with hematoxylin. Results: SARS-CoV-2-N-protein was detected by using a monoclonal antibody against the N-protein of SARS-CoV-2. The membranes of numerous syncytiotrophoblast cells revealed a strong, often homogeneous positivity. Additionally, single intervillous macrophages, Hofbauer cells and stroma cells were also found to express the SARS-CoV-2 N- protein. Placenta tissue of healthy triplets was completely negative (Figure 3). When qRT-PCR was performed from paraffin-embedded placenta we found a high Ct-value of 16 for the detection of the N-gene. In addition, the ACE2 receptor was stained by using the mouse anti-ACE2 antibody (Novusbio/R&D Systems, clone #171606, 1:2000) and the ultraView detection system (Ventana) mentioned above. ACE2 was found to be expressed in syncytiotrophoblasts, villous cytotrophoblasts, villous stromal cells, and some stromal cells and macrophages within the decidua, corresponding well to the SARS-CoV-2 infected cell types within the placenta (Figure 3).

## Discussion and Literature Review

### Congenital Infections With SARS-CoV-2

According to the classification by Shah et al. (6), there are different likelihoods (confirmed, probable, possible, unlikely, not infected) of different types of maternal-fetal-neonatal SARS-CoV-2 infection (congenital infection in liveborn neonate, congenital infection with fetal death, neonatal infection acquired intrapartum, neonatal infection acquired postpartum). We classified the triplets' infection as confirmed, congenital infections^1^ because immunohistology of the triplets' placenta revealed a strong membrane positivity for the viral N-protein of SARS-CoV-2 in the membranes of placental syncytiotrophoblast cells (Figure 3), as well as positivity of macrophages, Hofbauer cells and stroma cells. Thus, both the maternal side of the placenta and the fetal side were affected as Hofbauer cells are macrophages of fetal origin (7); syncytiotrophoblast cells are epithelial cells in direct contact with maternal blood (8). We did not analyse cord blood or amniotic fluid. The following aspects additionally support congenital infections: (1) delivery via C-section under maternal general anesthesia, minimizing the possibility of contact with virus particles from exhaled air; afterwards, immediate maternal quarantine until she tested negative twice and symptoms resolved completely; (2) positive tests in triplets I and III by PCR from swabs taken directly after birth and each triplet had at least two positive tests, making false-positive tests unlikely; (3) unusually thick oral secretions in all triplets after birth.

### Serologic Immune Response

The lack of a detectable immune response in the triplets directly after birth can be explained by the short time span between documented maternal infection and delivery (i.e., 1 week) as IgG production in pregnant women does not peak before 30 days after symptom onset (9). Additionally, Edlow et al. (10) reported that transplacental transfer of SARS-CoV-2 antibodies was lower than compared to influenza-HA-specific antibodies, suggesting inefficient transplacental transfer of antibodies against SARS-CoV-2. As well, a German study in 16 mothers with COVID-19 showed lower or the same antibody levels against SARS-CoV-2 in umbilical cord blood as compared to maternal blood (11). In accordance with the literature, our triplets I and III showed a seroconversion to IgG after 10 weeks; peaks in infant IgG production following perinatal infections were observed after 8 weeks in a term and in a preterm infant of 31 weeks (12). Whether the low IgG levels in our triplet II contributed to the child's severe course of disease is unclear; serum levels will be controlled at the next venipuncture indicated.

### COVID-19 in Multiple Pregnancies

Two published reports on COVID-19 in triplet pregnancies (13, 14) might raise concern for an elevated risk of VT in multiple pregnancies: A set of triplets was delivered via C-section at 30 5/7 weeks due to fetal deterioration and placental insufficiency. The heaviest triplet tested positive for SARS-CoV-2 on DOL 3 after an initially negative test (13, 15). Two triplets died due to sepsis and pmARDS. The SARS CoV-2 positive triplet required invasive ventilation as well but was later discharged in good health. No further tests were performed. Another set of triplets was born at 32 5/7 weeks by emergency C-section (14), due to maternal active labor and abnormal fetal presentation. All three tested positive by nasopharyngeal swabs on postnatal DOL 1 and 5. One triplet required respiratory support until DOL 3; otherwise, the neonates were asymptomatic. As neither amniotic fluid, nor the placenta or cord blood were evaluated, intrauterine transmission was suspected but could not be proven, either. Thus, currently available literature does neither provide consistent evidence for an elevated risk of congenital COVID-19 in multiple pregnancies, nor unequivocal proof of intrauterine transmission in the few cases suspicious for VT.

### Vertical Transmission

Previous reports on preterm neonates with COVID-19 are scarce: A Belgium 26-week preterm neonate contracted COVID-19 most likely through horizontal transmission on DOL 6. She received NIPPV prior to infection with SARS-CoV-2 and remained stable on CPAP support afterwards (2). A preterm born at 33 weeks reported as “possible VT” first tested positive by throat swab 16 h after birth, required 12 h of ventilation rather due to maternal sedation, and had a normal chest radiograph (16). A preterm of 32 weeks (17), born to a mother with a fatal course of COVID-19, fulfills the criteria for a “probable congenital infection” (6) due to detection of the virus in amniotic fluid but remained asymptomatic. In a UK neonatal SARS-CoV-2 cohort including 66 neonates (16 preterm) (5), two neonates were described with a “possible VT.” They had a positive nasopharyngeal swab 12 h after birth but received no further tests. In this cohort, incidence rates were higher in preterm compared to term neonates; yet, for preterm babies <32 weeks and <28 weeks, the confidence intervals were broad as they relied on only one case, respectively. One preterm neonate required invasive ventilation (at 36 weeks gestational age), indicating that most preterm neonates generally cope with SARS-CoV-2 infections well (5). This is supported by data from a cohort of 582 infected children identified through a European multicentre study including 25 countries (18): The neonatal subcohort (*n* = 35) comprised three preterm neonates, of which only one required mechanical ventilation- probably due to prematurity. Furthermore, one of the largest cohorts of neonates born to mothers with confirmed or suspected SARS-CoV-2 infection (19) (*n* = 100 mothers, *n* = 101 newborns) comprised 11 preterm babies, but there was neither evidence of a symptomatic neonatal infection nor of VT.

### Placental Inflammation by SARS-CoV-2

In a 35 week-preterm neonate, investigations showed an unequivocally confirmed transplacental infection with SARS-CoV-2 (20): The virus was detected in maternal and neonatal blood, in placental tissue, amniotic fluid and in multiple maternal and neonatal swabs. The child was delivered by urgent C-section, required resuscitation, and few hours of mechanical ventilation but showed no pulmonary manifestations thereafter. A cerebral MRI due to neurologic symptoms showed important white matter injury, estimated to be due to vascular inflammation caused by the SARS-CoV-2 infection (20). Pronounced inflammation due to SARS-CoV-2 has also been reported in two at-term placentas, both at the cytokine and at the genetic level (21). Placental SARS CoV-2 infection– as demonstrated e.g., by systematic investigation of 22 SARS CoV-2 positive placentas (22)- is consistently associated with chronic histiocytic intervillositis, trophoblast necrosis (22–24) and it is frequently accompanied by fibrin depositions (23, 25). These findings—chronic histiocytic intervillositis and trophoblast necrosis- were shown in both liveborn and stillborn neonates infected by transplacental transmission (24). The detection of complement C4d at the villous surface of infected placentas suggests a key role of the complement system in this process (25). It should also be noted that the extensive placental damage induced by SARS CoV-2 has caused pathologists to be concerned about potential neurologic sequelae in affected children (25). Schwartz et al. further showed that Hofbauer cell positivity is not required for transplacental fetal infections (22). Another group found that maternal clinical COVID-19 symptoms did not predict the severity of the SARS-CoV-2-related placental signature (26). In summary, currently available data emphasize the rarity of COVID-19 in preterm neonates, of a severe clinical course in preterm neonates associated with SARS-CoV-2, and of extremely rare in-utero-transmission of the virus.

### Role of Maternal Viremia

Our investigations point to the mechanism by which the virus was most likely transmitted to the children- maternal viremia- presuming presence of the virus in maternal blood already before delivery. As hypothesized by others (21), the virus probably spreads via the bloodstream to the uterus, and then infects the fetuses. Generally low and transient viremia in adults with symptoms of COVID-19 is estimated to occur in ~1% of patients (27, 28). A cohort study in adults showed higher plasma viral loads in critically ill as compared to less severely affected patients (29). Hitherto, viremia has not been investigated systematically in pregnant women with COVID-19: Numerous neonatal cases with suspected VT (3, 5, 16, 30, 31) did not investigate maternal viremia, nor was the subject addressed in a large number of pregnancies with uneventful neonatal outcomes (32–42). Hence, the significance of viremia in pregnant women is poorly understood. The Shah classification (6) also does not address the subject. In the cohort study conducted by Edlow et al. (10), there were no cases of viremia in maternal or in umbilical cord blood and no cases of VT. The authors concluded that lack of viremia in mothers may be protective against neonatal congenital infections (10). As an alternative mechanism for virus transmission, infection of amniotic fluid swallowed by the fetuses has been proposed (14).

### Relationship of Disease Severity With COVID-19

While our investigations have shown intrauterine virus transmission unequivocally, we cannot establish the nature of the relationship between the viral infection and disease severity with certainty (association vs. causality): Preterm neonates born at 29 weeks may require HFOV and/or circulatory support, irrespective of COVID-19. Thus, prematurity very likely contributed significantly to the observed clinical course. Yet, several factors [“Bradford-Hill criteria” for causality (43)]) argue in favor of an additional, major contributing role of COVID-19: (1) We observed a *biological gradient* between the triplets for viral load/duration of virus persistence on the one hand, and severity of clinical symptoms, laboratory and radiological findings on the other (“dose-response-relationship”); (2) *biological plausibility:* Abundant detection of viral RNA in throat swabs and tracheal aspirate, and production of unusually thick oral secretions make pulmonary manifestations of COVID-19 in the triplets plausible; (3) *coherence:* The observed pulmonary and radiologic findings are not contradictory to the pulmonary manifestations in older children or adults. Additionally, there are no other explanatory factors for a severe course of disease, such as missing fetal lung maturation, bacterial infections and/or long persistence of premature rupture of membranes.

## The Patients' Perspective

The triplets are followed-up in our Pediatric Neurodevelopmental Center. At corrected 9 months, triplet I developed horizontal nystagmus and showed moderate developmental delay; all investigations including cerebral MRI and electroencephalography were normal. Neurologic exam at follow-up was normal in triplets II and III and they have reached their developmental milestones according to their corrected age. Both parents are very supportive of the triplets.

We acknowledge some limitations: We did not evaluate maternal or neonatal samples for coronavirus mutations. We tested neither umbilical cord blood nor amniotic fluid for SARS-CoV-2 virus RNA/antibodies.

In conclusion, our report has several important clinical implications: (1) Counseling pregnant women should include to inform mothers about the rare possibility of a severe course of COVID-19 in (preterm) neonates. (2) Our triplets' cases show that vertical transmission of SARS-CoV-2, though rare, may occur as early as at the beginning of the third trimester. As new mutations have emerged, their pathogenicity with regard to pregnant women and their babies remains to be elucidated. (3) Published cases of affected neonates underline the importance of preventive measures, especially in high-risk expecting mothers. In this context, close follow-up is particularly advisable in mothers with multiple pregnancies. (4) Our observation of maternal viremia in association with severe neonatal COVID-19 warrants systematic investigation in larger cohorts of pregnant women in order to evaluate potential usability of maternal viremia as a marker to identify high-risk neonates in advance- as well as potential drivers for maternal viremia and in-utero virus transmission.

## Data Availability Statement

The raw data supporting the conclusions of this article will be made available by the authors, without undue reservation.

## Ethics Statement

Ethical review and approval was not required for the study on human participants in accordance with the local legislation and institutional requirements. Written informed consent from the participants' legal guardian/next of kin was not required to participate in this study in accordance with the national legislation and the institutional requirements. Written informed consent was obtained from the minor(s)' legal guardian/next of kin for the publication of any potentially identifiable images or data included in this article.

## Author Contributions

SD performed data collection, data interpretation, did the literature search, and drafted the manuscript. CA and SD conducted the microbial workup. TM and KK performed histopathologic workup including immunohistology. KA helped with the radiographic images. SW contributed to literature search, data interpretation, and critically reviewed the manuscript. TF, CA, KA, RZ, TM, KK, and AL contributed to data interpretation and critically reviewed the manuscript. FS contributed to data interpretation, critically reviewed the manuscript, and supervised the clinical management. SD, KA, and FS had full access to all clinical data and verified it. All authors approved the final version of the manuscript for submission.

## Conflict of Interest

The authors declare that the research was conducted in the absence of any commercial or financial relationships that could be construed as a potential conflict of interest.

## Publisher's Note

All claims expressed in this article are solely those of the authors and do not necessarily represent those of their affiliated organizations, or those of the publisher, the editors and the reviewers. Any product that may be evaluated in this article, or claim that may be made by its manufacturer, is not guaranteed or endorsed by the publisher.

## Footnotes

^1^Shah et al. (6) classify congenital infection as “confirmed” by detection of the virus in placental or fetal tissues only in fetal stillbirth. Yet, “unequivocal diagnosis of most fetal or neonatal infections is typically made by detection of the organism in culture or by nucleic acid amplifications tests [in amniotic fluid, fetal/neonatal blood or body fluid samples], or by histopathological demonstration of the organism in fetal/neonatal tissues”.

## Abbreviations

COVID-19: Coronavirus disease 2019
Ct: cycle threshold
CPAP: continuous positive airway pressure
CrP: C-reactive protein
DOL: day of life
HFOV: high-frequency oscillatory ventilation
NICU: neonatal intensive care unit
NIPPV: non-invasive positive pressure ventilation
PCR: reverse transcriptase polymerase chain reaction
pmARDS: premature acute respiratory distress syndrome
SARS-CoV-2: Severe Acute Respiratory Syndrome Coronavirus 2
SIMV: synchronized intermittent mandatory ventilation
VT: vertical transmission
WBC count: white blood cell count.

## References

[1] World Health Organization. WHO Coronavirus (COVID-19) Dashboard. (2021). Available online at: https://covid19.who.int/table (accessed November 4, 2021).

[2] Piersigilli F Carkeek K Hocq C van Grambezen B Hubinont C Chatzis O. COVID-19 in a 26-week preterm neonate. Lancet Child Adolesc Health. (2020) 4:476–8. 10.1016/S2352-4642(20)30140-1 32386562 PMC7252066

[3] Dong L Tian J He S Zhu C Wang J Liu C. Possible vertical transmission of SARS-CoV-2 from an infected mother to her newborn. JAMA. (2020) 323:1846–8. 10.1001/jama.2020.4621 32215581 PMC7099527

[4] Zeng H Xu C Fan J Tang Y Deng Q Zhang W. Antibodies in infants born to mothers with COVID-19 pneumonia. JAMA. (2020) 323:1848–9. 10.1001/jama.2020.4861 32215589 PMC7099444

[5] Gale C Quigley MA Placzek A Knight M Ladhani S Draper ES. Characteristics and outcomes of neonatal SARS-CoV-2 infection in the UK: a prospective national cohort study using active surveillance. Lancet Child Adolesc Health. (2021) 5:113–21. 10.1016/S2352-4642(20)30342-4 33181124 PMC7818530

[6] Shah PS Diambomba Y Acharya G Morris SK Bitnun A. Classification system and case definition for SARS-CoV-2 infection in pregnant women, fetuses, and neonates. Acta Obstet Gynecol Scand. (2020) 99:565–8. 10.1111/aogs.13870 32277845 PMC7262318

[7] Reyes L Golos TG. Hofbauer Cells: Their role in healthy and complicated pregnancy. Front Immunol. (2018) 9:2628. 10.3389/fimmu.2018.02628 30498493 PMC6249321

[8] Wang Y Zhao S. Vascular Biology of the Placenta. San Rafael, CA: Morgan and Claypool Life Sciences (2010). 21452443

[9] Kubiak JM Murphy EA Yee J Cagino KA Friedlander RL Glynn SM. Severe acute respiratory syndrome coronavirus 2 serology levels in pregnant women and their neonates. Am J Obstet Gynecol. (2021) 225:73.e1–e7. 10.1016/j.ajog.2021.01.016 33497654 PMC7825873

[10] Edlow AG Li JZ Collier A-RY Atyeo C James KE Boatin AA. Assessment of maternal and neonatal SARS-CoV-2 viral load, transplacental antibody transfer, and placental pathology in pregnancies during the COVID-19 pandemic. JAMA Netw Open. (2020) 3:e2030455. 10.1001/jamanetworkopen.2020.30455 33351086 PMC7756241

[11] Rathberger K Häusler S Wellmann S Weigl M Langhammer F Bazzano MV. SARS-CoV-2 in pregnancy and possible transfer of immunity: assessment of peripartal maternal and neonatal antibody levels and a longitudinal follow-up. J Perinat Med. (2021) 49:702–8. 10.1515/jpm-2021-0166 34116588

[12] Song D Prahl M Gaw SL Narasimhan SR Rai DS Huang A. Passive and active immunity in infants born to mothers with SARS-CoV-2 infection during pregnancy: prospective cohort study. BMJ Open. (2021) 11:e053036. 10.1136/bmjopen-2021-053036 34234001 PMC8264915

[13] Rabiei M Soori T Abiri A Farsi Z Shizarpour A Pirjani R. Maternal and fetal effects of COVID-19 virus on a complicated triplet pregnancy: a case report. J Med Case Rep. (2021) 15:87. 10.1186/s13256-020-02643-y 33602315 PMC7890395

[14] Alwardi TH Ramdas V Al Yahmadi M Al Aisari S Bhandari S Saif Al Hashami H. Is vertical transmission of SARS-CoV-2 infection possible in preterm triplet pregnancy? a case series. Pediatr Infect Dis J. (2020) 39:e456–8. 10.1097/INF.0000000000002926 33006879

[15] Schwartz DA Mohagheghi P Beigi B Zafaranloo N Moshfegh F Yazdani A. Spectrum of neonatal COVID-19 in Iran: 19 infants with SARS-CoV-2 perinatal infections with varying test results, clinical findings and outcomes. J Matern Fetal Neonatal Med. (2020) 1–10. 10.1080/14767058.2020.1797672 32783494

[16] Alzamora MC Paredes T Caceres D Webb CM Valdez LM La Rosa M. Severe COVID-19 during pregnancy and possible vertical transmission. Am J Perinatol. (2020) 37:861–5. 10.1055/s-0040-1710050 32305046 PMC7356080

[17] Zamaniyan M Ebadi A Aghajanpoor S Rahmani Z Haghshenas M Azizi S. Preterm delivery, maternal death, and vertical transmission in a pregnant woman with COVID-19 infection. Prenat Diagn. (2020) 40:1759–61. 10.1002/pd.5713 32304114 PMC7264605

[18] Götzinger F Santiago-Garcia B Fumadó-Pérez V Brinkmann F Tebruegge M. The ability of the neonatal immune response to handle SARS-CoV-2 infection. Lancet Child Adolesc Health. (2021) 5:e6–7. 10.1016/S2352-4642(21)00002-X 33484660 PMC7826031

[19] Dumitriu D Emeruwa UN Hanft E Liao GV Ludwig E Walzer L. Outcomes of neonates born to mothers with severe acute respiratory syndrome coronavirus 2 infection at a large medical center in New York City. JAMA Pediatr. (2021) 175:157–67. 10.1001/jamapediatrics.2020.4298 33044493 PMC7551222

[20] Vivanti AJ Vauloup-Fellous C Prevot S Zupan V Suffee C Do Cao J. Transplacental transmission of SARS-CoV-2 infection. Nat Commun. (2020) 11:3572. 10.1038/s41467-020-17436-6 32665677 PMC7360599

[21] Fenizia C Biasin M Cetin I Vergani P Mileto D Spinillo A. Analysis of SARS-CoV-2 vertical transmission during pregnancy. Nat Commun. (2020) 11:5128. 10.1038/s41467-020-18933-4 33046695 PMC7552412

[22] Schwartz DA Baldewijns M Benachi A Bugatti M Bulfamante G Cheng K. Hofbauer cells and COVID-19 in pregnancy. Arch Pathol Lab Med. (2021) 145:1328–328 10.5858/arpa.2021-0296-SA 34297794

[23] Schwartz DA Levitan D. Severe acute respiratory syndrome coronavirus 2 (SARS-CoV-2) infecting pregnant women and the fetus, intrauterine transmission, and placental pathology during the coronavirus disease 2019 (COVID-19) pandemic: it's complicated. Arch Pathol Lab Med. (2021) 145:925–8. 10.5858/arpa.2021-0164-ED 33878167

[24] Schwartz DA Baldewijns M Benachi A Bugatti M Collins RRJ de Luca D. Chronic histiocytic intervillositis with trophoblast necrosis is a risk factor associated with placental infection from coronavirus disease 2019 (COVID-19) and intrauterine maternal-fetal severe acute respiratory syndrome coronavirus 2 (SARS-CoV-2) transmission in live-born and stillborn infants. Arch Pathol Lab Med. (2021) 145:517ssio 10.5858/arpa.2020-0771-SA 33393592

[25] Watkins JC Torous VF Roberts DJ. Defining severe acute respiratory syndrome coronavirus 2 (SARS-CoV-2) placentitis. Arch Pathol Lab Med. (2021) 145:1341–9. 10.5858/arpa.2021-0246-SA 34338723

[26] Husen MF van der Meeren LE Verdijk RM Fraaij PLA van der Eijk AA Koopmans MPG. Unique severe COVID-19 placental signature independent of severity of clinical maternal symptoms. Viruses. (2021) 13:1670. 10.3390/v13081670 34452534 PMC8402730

[27] Lamouroux A Attie-Bitach T Martinovic J Leruez-Ville M Ville Y. Evidence for and against vertical transmission for severe acute respiratory syndrome coronavirus 2. Am J Obstet Gynecol. (2020) 223:91.e1–e4. 10.1016/j.ajog.2020.04.039 32376317 PMC7196550

[28] Wang W Xu Y Gao R Lu R Han K Wu G. Detection of SARS-CoV-2 in different types of clinical specimens. JAMA. (2020) 323:1843–4. 10.1001/jama.2020.3786 32159775 PMC7066521

[29] Chen L Wang G Long X Hou H Wei J Cao Y. Dynamics of blood viral load is strongly associated with clinical outcomes in coronavirus disease 2019 (COVID-19) patients: a prospective cohort study. J Mol Diagn. (2021) 23:10–8. 10.1016/j.jmoldx.2020.10.007 33122141 PMC7587132

[30] Hu X Gao J Luo X Feng L Liu W Chen J. Severe acute respiratory syndrome coronavirus 2 (SARS-CoV-2) vertical transmission in neonates born to mothers with coronavirus disease 2019 (COVID-19) pneumonia. Obstet Gynecol. (2020) 136:65–7. 10.1097/AOG.0000000000003926 32332320 PMC7219851

[31] Zeng L Xia S Yuan W Yan K Xiao F Shao J. Neonatal early-onset infection with SARS-CoV-2 in 33 neonates born to mothers with COVID-19 in Wuhan, China. JAMA Pediatr. (2020) 174:722–5. 10.1001/jamapediatrics.2020.0878 32215598 PMC7099530

[32] Lu D Sang L Du S Li T Chang Y Yang X-A. Asymptomatic COVID-19 infection in late pregnancy indicated no vertical transmission. J Med Virol. (2020) 92:1660–4. 10.1002/jmv.25927 32330313 PMC7264617

[33] Lv Y Gu B Chen Y Hu S Ruan T Xu G. No intrauterine vertical transmission in pregnancy with COVID-19: a case report. J Infect Chemother. (2020) 26:1313–5. 10.1016/j.jiac.2020.07.015 32859496 PMC7405770

[34] Liu D Li L Wu X Zheng D Wang J Yang L. Pregnancy and perinatal outcomes of women with coronavirus disease (COVID-19) pneumonia: a preliminary analysis. AJR Am J Roentgenol. (2020) 215:127–32. 10.2214/AJR.20.23072 32186894

[35] Li N Han L Peng M Lv Y Ouyang Y Liu K. Maternal and neonatal outcomes of pregnant women with coronavirus disease 2019 (COVID-19) pneumonia: a case-control study. Clin Infect Dis. (2020) 71:2035–41. 10.1093/cid/ciaa352 32249918 PMC7184430

[36] Pierce-Williams RAM Burd J Felder L Khoury R Bernstein PS Avila K. Clinical course of severe and critical coronavirus disease 2019 in hospitalized pregnancies: a United States cohort study. Am J Obstet Gynecol MFM. (2020) 2:100134. 10.1016/j.ajogmf.2020.100134 32391519 PMC7205698

[37] Chen H Guo J Wang C Luo F Yu X Zhang W. Clinical characteristics and intrauterine vertical transmission potential of COVID-19 infection in nine pregnant women: a retrospective review of medical records. Lancet. (2020) 395:809–15. 10.1016/S0140-6736(20)30360-3 32151335 PMC7159281

[38] Breslin N Baptiste C Gyamfi-Bannerman C Miller R Martinez R Bernstein K. Coronavirus disease 2019 infection among asymptomatic and symptomatic pregnant women: two weeks of confirmed presentations to an affiliated pair of New York City hospitals. Am J Obstet Gynecol MFM. (2020) 2:100118. 10.1016/j.ajogmf.2020.100118 32292903 PMC7144599

[39] Kuhrt K McMicking J Nanda S Nelson-Piercy C Shennan A. Placental abruption in a twin pregnancy at 32 weeks' gestation complicated by coronavirus disease 2019 without vertical transmission to the babies. Am J Obstet Gynecol MFM. (2020) 2:100135. 10.1016/j.ajogmf.2020.100135 32391520 PMC7206425

[40] Yang H Sun G Tang F Peng M Gao Y Peng J. Clinical features and outcomes of pregnant women suspected of coronavirus disease 2019. J Infect. (2020) 81:e40–4. 10.1016/j.jinf.2020.04.003 32294503 PMC7152867

[41] Zhang L Dong L Ming L Wei M Li J Hu R. Severe acute respiratory syndrome coronavirus 2(SARS-CoV-2) infection during late pregnancy: a report of 18 patients from Wuhan, China. BMC Pregnancy Childbirth. (2020) 20:394. 10.1186/s12884-020-03026-3 32641013 PMC7341473

[42] Zambrano LI Fuentes-Barahona IC Bejarano-Torres DA Bustillo C Gonzales G Vallecillo-Chinchilla G. A pregnant woman with COVID-19 in Central America. Travel Med Infect Dis. (2020) 36:101639. 10.1016/j.tmaid.2020.101639 32222420 PMC7271224

[43] Rothman KJ Greenland S Lash TL. Modern Epidemiology. 3rd ed. Philadelphia, PA, Baltimore, MD, New York, NY: Wolters Kluwer Health Lippincott Williams & Wilkins (2008).

